# Grand Challenges in Microbiotechnology: Through the Prism of Microbiotechnology

**DOI:** 10.3389/fmicb.2020.00430

**Published:** 2020-03-20

**Authors:** Eric Altermann, William J. Hickey

**Affiliations:** ^1^AgResearch, Palmerston North, New Zealand; ^2^Riddet Institute, Massey University, Palmerston North, New Zealand; ^3^Department of Soil Science, University of Wisconsin, Madison, WI, United States

**Keywords:** microbiotechnology, microbial community, bioremediation, microbial ecology, biodegradation, metabolic engineering, protein function, microbial consortia

## Introduction

Microbes have conquered almost every conceivable space on earth—from high atmospheres to terrestrial and aquatic ecosystems to extreme places such as geothermal vents in the deep sea, oil reservoirs, or boiling hot springs. Survival in these varied environments necessitates a breathtaking span of genetic diversity, enabling the metabolism and synthesis of many different substrates for both energy creation and biomass buildup and to gain an evolutionary advantage over other life forms sharing the same ecosystem. Of particular biotechnological interest are molecules referred to as secondary metabolites that often feature a unique chemical makeup and can encompass functions such as ion scavenging, quorum sensing, or act as antimicrobials.

With the advent of anthropogenic impacts on our planet, such as the change or creation of new ecosystems (e.g., waste water treatment plants, large scale commercial fermentation processes) or the deposition of novel compounds and toxic pollutants into the environment, microbes have shown remarkable adaptability to utilize these newly introduced materials as novel sources of energy.

It is this surprisingly vast and adaptable biochemical potential of microbes that we have come to realize and exploit for specific tasks ranging from fermentation processes modifying material properties, to the manufacture of high-value stereospecific chemicals and polymers, to the breakdown of hazardous substances.

The application of microbes to industrial processes is commonly known as Microbiotechology. Under this umbrella, many different subareas are combined and have been explored over the last decade in Frontiers in Microbiology specialty section on “Microbiotechnology” (previously “Microbiotechnology, Ecotoxicology and Bioremediation”).

## Moving Research Over a Decade

Since 2011, Micro Bio Technology (MBT) has been dedicated to offer a platform for high quality research, investigating the exploitation of microbial genetic diversity toward environmental and industrial applications. How did research push the boundaries of our knowledge and understanding of microbial processes in nature and their biotechnological application?

**Figure 1:**
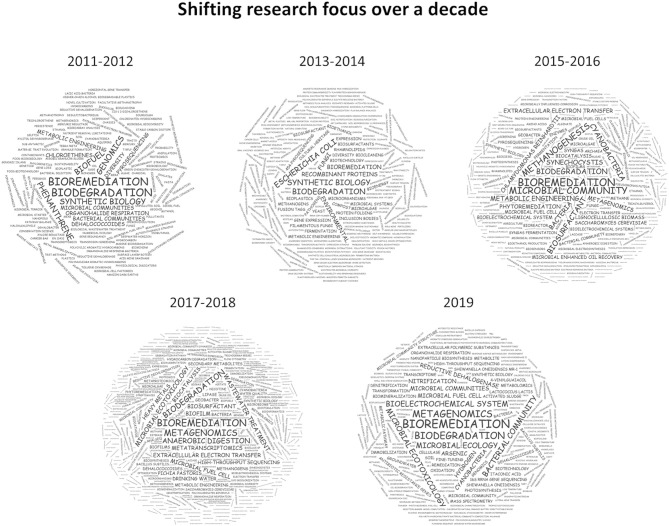
Weighted keyword clouds of 800 articles published between 2011 and 2019 in MBT. While “bioremediation” and “biodegradation” feature prominently in all years, a clear trend toward multi-omics technologies can be observed since 2015.

Bioremediation via methanotrophs to effect aerobic degradation of chlorinated hydrocarbons, was one of the most cited publications in the first year of this specialty (Semrau, 2011). Chlorinated hydrocarbons are widely used as plastics, solvents, insulators, or pesticides, they are now ubiquitous in the environment and the ecotoxicity of some of these (e.g., dioxins, DDT) have particular concerns in environmental health and toxicology. The focus on biodegradation and bioremediation was further elaborated by a perspective on how to quantify biodegradation of substances from a regulatory perspective (Thouand et al., 2011)and the development of genetic tools to improve biodegradation by specific microbes (Hickey, 2011). Following this line of research, publications emerged targeting the biodegradation of many different chemical classes through classical genomics approaches and the characterization of microbial communities (Figure 1, 2011–2012). In the following 2 years, a notable shift was observed toward synthetic biology, microbiotechnology, and metabolic engineering driven by an increasing genome space (Figure 1, 2013–2014) (De Gannes et al., 2013; Moe-Behrens et al., 2013; Elena et al., 2014; Rosano and Ceccarelli, 2014).

From 2015 onwards, the progressive drop in DNA sequencing cost enabled the investigation of microbial communities in much greater detail and a prominent research area on microbial communities was established and more recently consolidated under “metagenomic” research. But not only the community structure saw a much more in-depth exploration, the ready availability of draft genome sequences of individual microbes and new gene and genome editing tools enabled substantial advances in metabolic engineering. In particular the exploitation of microbial molecule and polymer synthesis such as exopolysaccharides, pigments, lipids, or biosurfactants has received much attention (Figure 1, 2015–2018) (Rosano and Ceccarelli, 2014; Antoniou et al., 2015; Bonugli-Santos et al., 2015; Giddings et al., 2015; Ishii et al., 2015; Kracke et al., 2015; Moscovici, 2015; Schmid et al., 2015; Torino et al., 2015; Uzoigwe et al., 2015; Whitfield et al., 2015; Zeldes et al., 2015; De Almeida et al., 2016; Ghosal et al., 2016; Gill et al., 2016; Gouda et al., 2016; Jugder et al., 2016; Liew et al., 2016; Minhas et al., 2016; Santiago et al., 2016; Shchegolkova et al., 2016; Singh et al., 2016; Thijs et al., 2016).

Most recently, the realization that a comprehensive understanding of biological ecosystems as a whole is key to more advanced biotechnological approaches has led to an even stronger integration of ‘omics technologies into microbial ecology, interrogating bacterial communities for their mechanistic function toward bioremediation and biodegradation (Figure 1, 2019) (Abbas et al., 2019; Carrillo-Barragan et al., 2019; Chonova et al., 2019; Crouzet et al., 2019; Cycon et al., 2019; Jacquin et al., 2019; Jaiswal et al., 2019; Kantor et al., 2019; Li F. et al., 2019; Li J. et al., 2019; Marco and Abram, 2019; Meng et al., 2019; Miller et al., 2019; Pinnell and Turner, 2019; Ragab et al., 2019; Rocca et al., 2019; Sengupta et al., 2019; Suri et al., 2019; Xiao et al., 2019; Xu et al., 2019).

A developing area of microbiotechnology “phytoremediation” was defined as “the synergistic actions of plants and microorganisms […] to clean up soils [and other ecosystems]” (Thijs et al., 2016). While the independent investigation of different microbes or plants has been investigated for bioremediation or the production of compounds, the synergistic interactions between both agents is key to understand and fully exploit their potential for both biotechnical applications and persistence. Therefore, more research into holistic, mechanistic examinations of entire ecosystems will be required moving into the future.

## Moving Into the Future of Microbiotechnology: Grand Challenges

‘OMICs technologies have an ever more prominent presence in publications in all areas of microbiotechnology and their impact on accelerating our knowledge framework can hardly be overstated. ‘OMICS technologies were initially often thought of as only DNA-based technologies, such as high-throughput genome sequencing, metagenomics and to a smaller degree, metatranscriptomics. With the advent of high-throughput approaches in other disciplines, ‘omics technologies now also include metaproteomics, metabolomics, epigenetics, and phenomics, alongside other emerging ‘omics studies. However, the common pitfall of using these high-throughput technologies is to utilize them as a mere looking glass. This one-dimensional approach has led to a large number of descriptive science publications that, while delivering new insights into microbial community composition and their changes in response to time or stressors, lack true mechanistic insights on community and designed consortia levels.

The functional link between *in silico* ‘OMICS technologies and *in vitro*/*in vivo* engineering, experiments, validation, and applications of individual microorganisms and strategically designed consortia holds many grand challenges, two of which we envision are a Grand Challenge in (1) Integrative Molecular Analysis (IMA) and (2) Microbial Community Mechanisms (MCM).

A first dimension of the IMA Grand Challenge will be to gain an understanding of how changes in the amounts and types of cellular molecules (e.g., mRNA, proteins) are inter-related and how the collective dynamics of these molecules is ultimately manifested in cellular activities. Achieving this goal would be a quantum leap in fundamental microbiology and, consequently, enable attendant advances in applied microbiological fields ranging from design of biosynthetic pathways to managing microbes in the environment.

A key hurdle in attaining this goal is the mismatch between the linear approaches used to study the molecules that mediate microbial processes and the non-linear mechanisms through which these molecules mediate cellular behaviors. Currently, classes of key molecules are studied individually, removed from the cells and all other molecules with which they would natively interact to create dynamic systems. For example, detection and quantification of mRNA (via hybridization array, RNAseq, or qPCR) is widely used as a proxy for cognate protein levels (and by extension, an activity/phenotype of interest), yet there is ample evidence that no such strict correspondence exists between these molecules. Instead, types and levels of cellular proteins and metabolites (and hence phenotypes) are the result of numerous types and levels of control. Furthermore, the importance of any one of these regulatory controls may vary between organisms or within an organism depending on the particular protein or condition of the cell.

Obtaining a more rigorous understanding of the cellular processes that control microbial life will require a dramatic revolution in the technologies and approaches applied in molecular studies. Advanced technologies are needed that enable multiple molecule types to be queried simultaneously, ideally within cells. This grand challenge in technological development lies at the nexus of microbiology with many other fields including chemistry, biochemistry and biophysics, and will be a truly interdisciplinary effort.

Meeting the IMA Grand Challenge will require a second dimension by advancing technologies and methodologies for deciphering gene function. This is a multifaceted issue and encompasses investigation of gene function in cultured organisms as well as functions for those that exist only *in silico* and originate in genome sequence derived from environmental DNA extracts. For cultured organisms, genetic manipulations that are essential to decipher, or alter, functions of gene products have not advanced at the same rapid pace as other nucleic acid technologies and represent a key bottleneck in efforts to deepen insights into microbial biology.

Some relatively recent developments in genetic engineering, such as recombineering and CRISPR-based approaches, circumvent some of the limitations of conventional methods. But, as with the conventional approaches, their application to any given organism typically requires resolution of many issues through empirical testing, which effectively restricts the development and application to a relatively small number of laboratories. Thus, achievement of this Grand Challenge would be facilitated by the development of centralized efforts within government institutions that focus on the development and dissemination of technologies in partnerships with academia.

A third dimension to the IMA Grand Challenge is deciphering biological roles for the large and ever-expanding database of genes with unknown function. These efforts rely heavily on computational approaches for function prediction, and improvements in computation will be essential to advance this field. Specifically, to discover truly novel functions, a revolution in computational methods is needed that would allow *de novo* assignment of function with little, or no, reference to information in existing databases. Computational methods are also needed that include molecular interactions in function prediction, since the activities of the majority of proteins likely arise from such processes. Advancements have already been made in predicting protein-protein interactions, and these need to be improved and expanded to include the diversity of molecules that exist within the cell. Beyond the computational challenges in identifying putatively novel functions, there are also advancements needed in genetic systems (see above) that would be needed to prove function and provide a means for translating the potential of novel genes into products or functions.

The MCM Grand Challenge centers on obtaining comprehensive mechanistic understanding on how microbial communities interact with each other, with potential hosts and with the environment. It is clear that microbes in isolation only display a fraction of their genetic potential and gains achieved by understanding and exploiting synergistic interactions could revolutionize microbiotechnolgy in all its aspects. Such understanding can only be achieved by utilizing and further developing gene and genome engineering tools alongside the plethora of ‘omics technologies. Similar to the synergistic play of multiple microbes together, we must develop new systems and technological infrastructure to successfully mature, combine, interrogate and visualize the high-dimensional ‘OMICS data so that they can inform mathematical models and aid in strategically select gene candidates for functional investigation and the deliberate assembly of microbial consortia to achieve a biotechnological goal. The MCM Grand Challenge will not be able to be achieved through the scope of Microbiotechnology alone—it will require a truly inter-disciplinary collaboration across many science disciplines.

The extent to which research in microbiotechnology can be translated into novel societal uses will be dependent upon the depth to which these microbial systems are understood. Thus, revolutionary advancements in how, or what, microbial processes are employed to achieve some goal must be preceded by advancements in fundamental science such as those presented above. These advancements would move biosciences well beyond the current age of ‘OMICs, technologies, which have been essential in the evolution of biosciences, but which lack the multidimensionality that will be needed in the next generation of analytical tools to gain deeper insights into cellular functions and, consequently, develop new ways in which the potential of microbial systems is captured.

## Author Contributions

EA and WH have devised and written the Grand Microbiotechnology Challenge.

### Conflict of Interest

The authors declare that the research was conducted in the absence of any commercial or financial relationships that could be construed as a potential conflict of interest.
